# High-Density Polyethylene Custom Focusing Lenses for High-Resolution Transient Terahertz Biomedical Imaging Sensors

**DOI:** 10.3390/s24072066

**Published:** 2024-03-24

**Authors:** Debamitra Chakraborty, Robert Boni, Bradley N. Mills, Jing Cheng, Ivan Komissarov, Scott A. Gerber, Roman Sobolewski

**Affiliations:** 1 Materials Science Program, University of Rochester, Rochester, NY 14627-0166, USA; dchakrab@ur.rochester.edu (D.C.); jcheng32@ur.rochester.edu (J.C.); 2 Laboratory for Laser Energetics, University of Rochester, Rochester, NY 14623-1299, USA; rbon@lle.rochester.edu (R.B.); ikom@lle.rochester.edu (I.K.); 3 Department of Surgery, University of Rochester Medical Center, Rochester, NY 14642-0001, USA; bradley_mills@urmc.rochester.edu (B.N.M.); scott_gerber@urmc.rochester.edu (S.A.G.); 4 Department of Electrical and Computer Engineering, University of Rochester, Rochester, NY 14627-0231, USA; 5 Department of Physics and Astronomy, University of Rochester, Rochester, NY 14627-0171, USA

**Keywords:** terahertz, terahertz-time-domain spectroscopy, high-resolution THz imaging, lens design, aspheric lens, high-density polyethylene lens material, terahertz medical imaging of tissue malignancies

## Abstract

Transient terahertz time-domain spectroscopy (THz-TDS) imaging has emerged as a novel non-ionizing and noninvasive biomedical imaging modality, designed for the detection and characterization of a variety of tissue malignancies due to their high signal-to-noise ratio and submillimeter resolution. We report our design of a pair of aspheric focusing lenses using a commercially available lens-design software that resulted in about 200 × 200-μm^2^ focal spot size corresponding to the 1-THz frequency. The lenses are made of high-density polyethylene (HDPE) obtained using a lathe fabrication and are integrated into a THz-TDS system that includes low-temperature GaAs photoconductive antennae as both a THz emitter and detector. The system is used to generate high-resolution, two-dimensional (2D) images of formalin-fixed, paraffin-embedded murine pancreas tissue blocks. The performance of these focusing lenses is compared to the older system based on a pair of short-focal-length, hemispherical polytetrafluoroethylene (Teflon^TM^) lenses and is characterized using THz-domain measurements, resulting in 2D maps of the tissue refractive index and absorption coefficient as imaging markers. For a quantitative evaluation of the lens effect on the image resolution, we formulated a lateral resolution parameter, *R*_2080_, defined as the distance required for a 20–80% transition of the imaging marker from the bare paraffin region to the tissue region in the same image frame. The *R*_2080_ parameter clearly demonstrates the advantage of the HDPE lenses over Teflon^TM^ lenses. The lens-design approach presented here can be successfully implemented in other THz-TDS setups with known THz emitter and detector specifications.

## 1. Introduction

The terahertz (THz) region (~0.3- to 30-THz spectral range) of the electromagnetic radiation spectrum is considered the “last frontier” in electromagnetics because it lies between the optics and radio-frequency engineering domains [1]. THz radiation has received notable interest due to its widespread applications, ranging from ultrafast wireless communications through security inspection to biosensing. Features like a submillimeter spatial resolution, femtosecond time resolution, and high signal-to-noise ratio (SNR) are the main reasons THz imaging has become attractive as a noninvasive probing tool [2]. Another principal advantage of THz electromagnetic radiation is its ability to propagate through various materials, allowing for a broad application of completely noninvasive, contactless imaging with applications including airport security scanning [3], package-integrity pharmaceutical testing [4], food quality and safety monitoring [5], art and culture conservation [6], astronomy and space observation [7], and biomedical spectroscopy and imaging [8]. Moreover, this label-free imaging modality offers a “molecular fingerprint” of chemical and biological materials in the previously unexplored electromagnetic spectrum since the photon energy of the THz wave (~1 to 100 meV) primarily matches energy levels related to low-frequency motions, including molecular skeletal vibration, rotation, and translation [9]. As a result, diverse biomolecules can be effectively detected and defined using their unique spectral fingerprints. THz time-domain spectroscopy (THz-TDS) has become popular tool since the measurement scheme preserves both the magnitude and phase of the measured spectra; hence, the material’s dielectric properties as a function of the frequency under investigation can be characterized directly from experimentally obtained time-domain signals without employing any complicated data analysis pipeline, such as solving the Krames–Kronig equations, unlike in other spectroscopic techniques like Fourier transform infrared spectroscopy [10].

With the advancement of THz imaging, a vast number of research articles have focused on novel THz emitters, including photoconductive antennae, spintronic emitters, quantum cascade lasers, resonant tunnel diodes, and even superconducting sources [11,12,13], as well as detection systems that include direct photoconductive antennae and heterodyne systems based on superconductor–insulator–superconductor junctions or superconductor hot-electron nano-bolometers [14]. However, the literature addressing the need for improved metrology to enhance the spatial resolution of THz-TDS imaging systems is limited. Most THz-TDS setups contain off-axis parabolic mirrors for focusing a THz beam [15]. The latter, however, comes with significant drawbacks, like a complex alignment with six degrees of positioning freedom, an inconvenient zigzag optical path, a diffraction-limited field of view, an inability to correct for spherical aberrations, a high cost, and, finally, the tendency to have a bulky optical setup. Since achieving and maintaining precise alignment is essential, they might not be the best choice for applications with size and weight constraints, especially for wide-angle THz beams. On the other hand, using lenses to focus THz beams can offer several advantages compared to off-axis-parabolic mirrors, including simplicity in setup, correctable spherical aberrations, cost-effectiveness, compactness, a wider field of view, easy customization, and reduced alignment sensitivity.

Among the commercially available THz beam guiding lenses, the most common choice is hyperhemispherical and elliptic silicon lenses designed to collimate or focus THz beams [16]. However, polymer-based lenses can be a cost-effective alternative to expensive Si lenses. Polymer-based lenses, fabricated from polymethylpentene (TPX^TM^), polytetrafluoroethylene (Teflon^TM^), or high-density polyethylene HDPE with plano-convex or plano-convex-aspheric curvatures, are commonly employed to focus THz beams. Notably, among these three polymers, HDPE (average refractive index 1.54) exhibits the highest average refractive index in the 0.1–3 THz range, making it more suitable for use as the THz lens material compared to TPX^TM^ (average refractive index 1.46) and Teflon^TM^ (average refractive index 1.40) [17]. Accordingly, HDPE has less dispersion compared to TPX^TM^ and Teflon^TM^ [17]. Furthermore, a comparative analysis of absorption characteristics reveals that HDPE and TPX^TM^ exhibit lower absorption than Teflon^TM^, with TPX^TM^ exceeding HDPE’s absorption below ~1 THz, while being lower above 1 THz [17]. A further concern with TPX^TM^ is that the high shrinkage ratio causes limited yield during injection molding, which can increase the cost of the TPX^TM^ lens compared to HDPE [18]. Consequently, considering the collective attributes of refractive index, dispersion, and absorption, HDPE can be chosen as the optimal choice among the polymer options for THz lens material. Since commercially available lenses come with a fixed curvature and focal length, a custom HDPE lens can help tailor the optical performance and provide a desired form factor without altering the existing setup.

In this article, we describe an HDPE plano-aspheric lens-design procedure for incorporation into an existing THz-TDS system in transmission geometry, including commercial photoconductive antennae mounted with in-built hyper-hemispherical Si (HH-Si) lenses. The system based on the HDPE lenses is compared to our older version, used in [10], and based on two hemispherical, short-focal-length Teflon^TM^ lenses. To assess the performance of the HDPE lenses, we performed imaging of murine pancreas tissue samples, and present the resulting high-resolution, two-dimensional (2D) maps of the tissue refractive index *n* and absorption coefficient *α* imaging markers. Additionally, we introduce a lateral resolution calculation parameter called *R*_2080_ in order to evaluate the performance of our THz-TDS imaging system with HDPE lenses, as well as to directly compare it to the THz-TDS system with Teflon^TM^ lenses.

## 2. Materials and Methods

### 2.1. Choice of Material for Aspheric Lens Design

**Figure 1:**
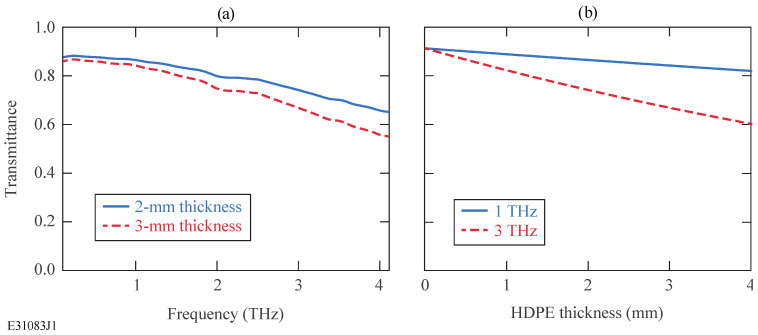
(**a**) Transmittance (including surface reflections) with respect to THz frequency for an HDPE material; (**b**) THz transmittance (including surface reflections) with respect to HDPE material thickness. The data for these plots are adopted from [17,19].

We chose HDPE (McMaster-Carr, Chicago, IL, USA)—a low-cost, chemical-resistant polyethylene with a density above 0.95 g/cm^3^—to craft our THz focusing lenses since it can be easily machined and its transmittance in the 0.1–4-THz frequency range (the bandwidth of our THz-TDS system) is always above 60%, as is shown in Figure 1a, [17,19]. In addition, Figure 1b demonstrates the high THz transmittance through HDPE with respect to its thickness [17,19]. Moreover, lenses made from HDPE are achromatic since the material has an almost constant index of refraction across the 0.1–4-THz bandwidth. HDPE also has high rigidity, which makes it suitable for use in, e.g., cryostat windows. Finally, HDPE has a low dielectric constant of ~2.36 over the entire 4–300 K temperature range, which ensures low insertion loss [17,19].

### 2.2. Design of Plano-Aspheric Lenses

**Figure 2:**
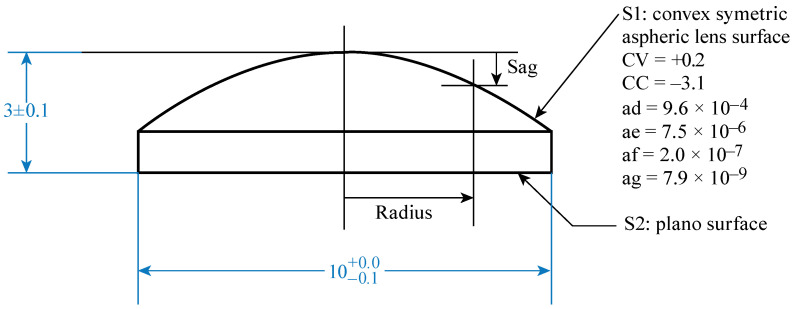
Schematic of a plano-aspheric lens. All dimensions are in mm.

We designed a pair of identical plano-aspheric lenses using a lens-design software called optics software for layout and optimization (OSLO version 24.1.0) [20]. OSLO enabled us to analyze our design and subsequently empirically test that our lenses can obtain a small focal spot with high spatial resolution. The geometry of the aspheric lens is provided in Figure 2.

Aspheric surfaces are slightly altered from spherical surfaces to reduce spherical aberrations, which causes incident light rays to focus on different spots while forming an image, resulting in a blur, an inherent phenomenon that is typical for spherical lenses. By altering the conic constant and aspheric coefficients of the lens’s curved surface, an aspheric lens can be built to minimize aberration, which not only increases the image quality but also minimizes the number of necessary optical elements. For an aspheric lens surface, to optimize its performance, we used the following equation [21]:

(1) $$Z_{\mathrm{Sag}}\left(r\right)=\frac{\mathrm{CV}r^{2}}{1+\sqrt{1-{\mathrm{CV}}^{2}\left(\mathrm{CC}+1\right)r^{2}}}+\mathrm{ad}r^{4}+\mathrm{ae}r^{6}+\mathrm{af}r^{8}+\mathrm{ag}r^{10},$$

where the *z* axis denotes the optic axis; *Z*_Sag_(*r*) is the Sag, i.e., the *z* component of the surface’s displacement from the vertex at *r* distance from the axis (see Figure 2); CV denotes the curvature of the vertex; CC stands for the conic constant; and, finally, the aspheric coefficients ad, ae, af, and ag determine the deviation of the surface from the axially symmetric quadric surface. After optimization, CV, CC, ad, ae, af, and ag are the optimized coefficients.

### 2.3. Fabrication of Plano-Aspheric Lenses

**Figure 3:**
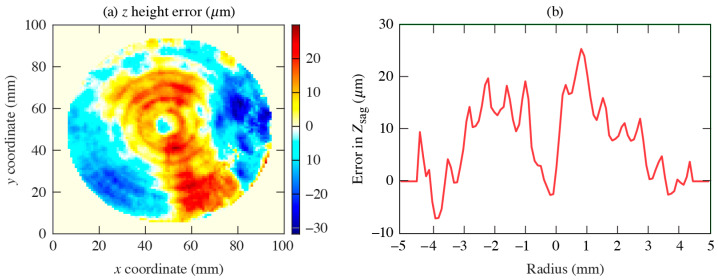
(**a**) Surface profile map of the aspheric *x*–*y* surface of one of the fabricated lenses with reference to the ideal designed aspheric surface. (**b**) Line plot of the surface error along the lens radius.

Our lenses were fabricated in our in-house machine shop using a standard, computer-controlled lathe for regular machining operations [22]. The lenses were then characterized using an optical profilometer to measure the surface roughness and profile. The surface profile map of the *x–y* aspheric surface of one of our fabricated lenses is shown in Figure 3a. It was generated using the ideal aspheric design as a reference. The line plot in Figure 3b denotes the error in *Z*_Sag_, i.e., the deviation from the ideal design along with the radius of the lens. We note that the measured deviation is minimal, of the order of 20 μm or less.

### 2.4. Integration of the Lens Pair into THz-TDS Setup

**Figure 4:**
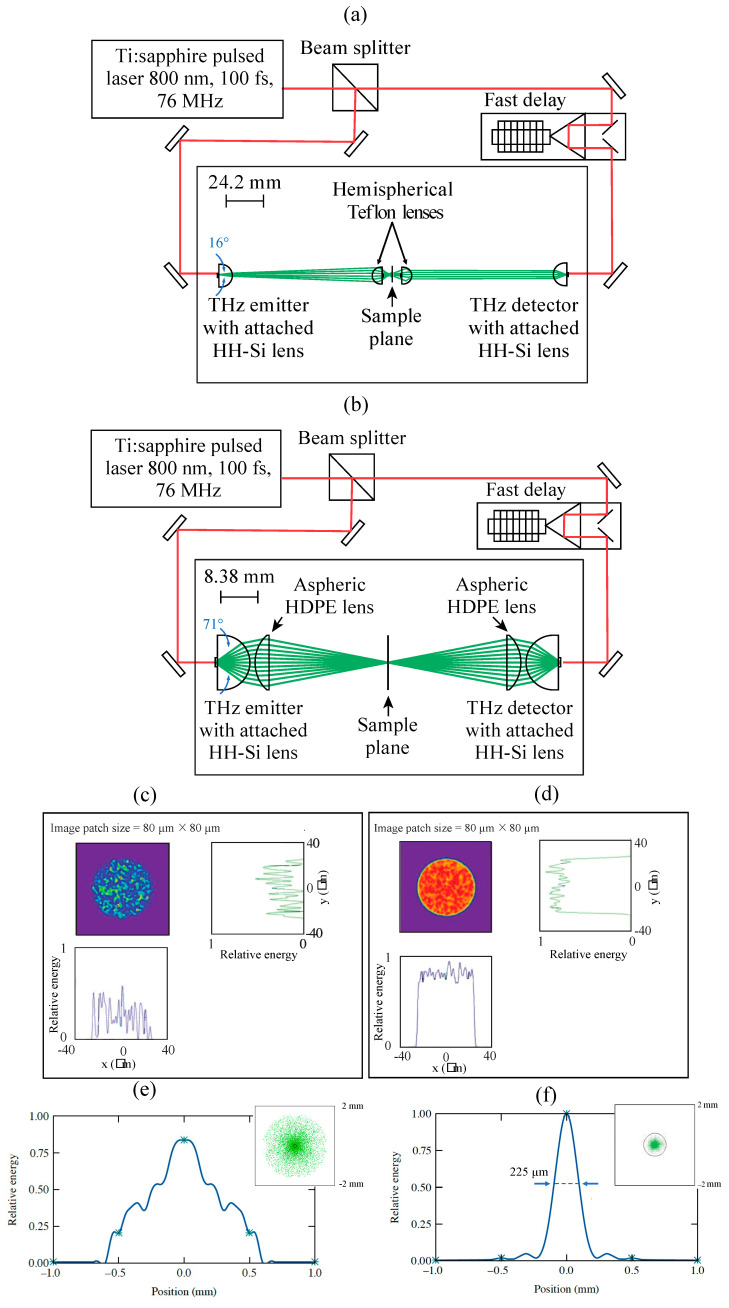
(**a**) Schematic diagram of the THz-TDS setup using the pair of hemispherical Teflon^TM^ lenses. Optical pulses of 100 fs wide, with an 800 nm wavelength were generated by a commercial Ti:Sapphire laser and were split using a 50:50 polarizing beam-splitter. The pump beam was used to excite a low-temperature grown GaAs (LT-GaAs) THz emitter, whereas the probe beam was used to excite an LT-GaAs detector antenna. The emitted THz beam is passed through an HH-Si lens and a hemispherical Teflon lens to focus the signal on a sample plane. Another Teflon^TM^ lens and an HH-Si lens attached to the detector antenna focus the beam onto the detector. (**b**) Schematic diagram of the optical system using the pair of aspheric HDPE lenses instead of Teflon^TM^ lenses. A custom-made plano-aspheric HDPE lens tightly focuses the THz beam from the HH-Si lens attached to the THz emitter. After that, the diverging beam from the focal point is collimated/focused by the second HDPE lens. The rays are then passed through the HH-Si lens attached to the detector to focus it on the LT-GaAs photoconductive antenna detector (the rightmost element). (**c**) <1% energy coupling from the 50 µm diameter emitter to the 50 µm diameter detector using a hemispherical Teflon^TM^ lens. The irradiance at the center of the detector is 74%. The cross-section line scans at a normalized energy scale are provided along both the *x* and the *y* axes. The ray-trace results were generated by launching 1.00 × 10^6^ rays, of which 1.25 × 10^3^ passed and 0.125% power was transmitted. (**d**) An energy coupling of >90% energy was passed from the 50 µm diameter emitter to the 50 µm diameter detector using an aspheric HDPE lens. The irradiance occurs at the center if the detector is at 74%. The cross-section line scans on a normalized energy scale are provided along both the *x* and the *y* axes. The ray-trace results were generated by launching 1.00 × 10^5^ rays, of which 9.08 × 10^4^ passed and 90.8% power was transmitted. (**e**) A 600 µm diameter spot at the focal point (sample plane) using a hemispherical Teflon^TM^ lens. The inset shows a spot diagram, resulting in about 300 × 300 μm^2^ focal spot size corresponding to 1 THz central frequency. A very small back circle at the center indicates the diffraction limit (also known as the ‘Airy disk’) of 76 µm. The green dots represent rays originating on the axis location of the emitter, traced geometrically by refraction at the lens surfaces. The geometrical rms radius is found to be 534 µm. Hence the system is not diffraction-limited. (**f**) A 200 µm diameter spot at the focal point (sample plane) aspheric HDPE lens. The inset shows a spot diagram at the focal point using the aspheric lenses, resulting in about a 100 × 100-μm^2^ focal spot size corresponding to 1 THz central frequency. The back circle at the figure center indicates the diffraction limit (also known as the ‘Airy disk’) of 565 µm. The geometrical rms radius is found to be 225 µm, which is the full-width-at-half-maximum (FWHM) of relative energy vs. position diagram. Hence, the HDPE system is diffraction-limited.

We used 100 fs wide, 800 nm wavelength optical pulses generated by a commercial Ti:Sapphire laser (Coherent, Santa Clara, CA, USA) to excite a low-temperature grown GaAs (LT-GaAs) THz emitter, as well as probe an LT-GaAs detector antenna (TeraVil Ltd., Vilnius, Lithuania). Additionally, both the antennae were equipped with adjustable HH-Si lenses mounted immediately in front of them to collimate the THz beam. We performed our previous experiments by placing a pair of short-focal-length hemispherical Teflon^TM^ lenses to focus the emitted THz beam onto the sample plane and collect the transmitted signal, as presented in Figure 4a [10]. In the present work, a pair of aspheric lenses were designed considering our existing THz-TDS setup in terms of transmission geometry. We then realigned the same setup, replacing the Teflon^TM^ lenses with the aspheric HDPE lenses, as shown in Figure 4b. As shown in Figure 4a,b, the THz rays after passing the HH-Si lenses are somewhat collimated, but there is also a significant number of spherical aberrations, which is a typical signature of the spherical surface. The aspheric lens (Figure 4b) then not only focuses the beam onto a focal point where the sample is placed, but also corrects the spherical aberration caused by the Si lens. (We note that off-axis parabolic mirrors cannot correct for this type of spherical aberration.) After traveling through the sample, a diverged beam is then collected by an identical aspheric lens to be collimated and then focused by the HH-Si lens integrated with the detector. The advantage of using the aspheric HDPE lenses compared to Teflon^TM^ lenses is two-fold. Firstly, the HDPE lenses provide more throughput from the emitter to the detector. In the Teflon^TM^ lens system, we could only collect 16° of the full-angle emission (Figure 4a), whereas using HDPE lenses enabled us to collect 71° full-angle emission (Figure 4b). Also, only 0.12% power is transmitted to the detector while using the Teflon^TM^ lenses, as shown in Figure 4c, whereas 90.82% power is transmitted to the detector using HDPE lenses, as shown in Figure 4d. Secondly, the aspheric HDPE lens system enables a smaller sampling spot size of 200 μm, as shown in Figure 4e, compared to the 600 μm spot size (Figure 4f), using the hemispherical Teflon^TM^ lens system. The inset of Figure 4f shows a spot diagram at the focal point (sample plane) using the aspheric HDPE lens system, indicating a very tightly focused beam with a geometrical root–mean–square (rms) radius of about 225 μm, which is significantly less than the diffraction limit of 565 μm (the black circle). Therefore, the spatial resolution is limited only by diffraction.

For THz-TDS studies, we mounted the sample onto a stepper motor-controlled *x–y* stage to perform raster scans for imaging. We used a purged nitrogen box to remove atmospheric water features. THz transients were detected with a registering system containing a fast optical delay line that allowed for the collection of signals with a 1.78 fs time resolution and 1000 averages within a less than 1 min long window. This THz registration system eliminated the need for a conventional combination of a slow stepper motor-controlled delay line and a lock-in amplifier. We obtained an SNR of 86.24 dB using the above setup. However, it takes only 10–15 s to obtain a 1000-point average time-domain waveform with an SNR of 64 dB. The details of our experimental setup can be found in [10]. The tested tissue was raster-scanned with a 100 μm step size in both *x* and *y* directions to generate 2D *n* and *α* as imaging maps within a usable 0.1–4-THz bandwidth.

### 2.5. Data Processing Pipeline Using a Maximum a-Posteriori Probability Estimation Procedure

The *n* and *α* data were reconstructed from the THz time-domain signals measured at each pixel location, using a maximum *a-posteriori* probability (MAP) estimation procedure described in detail in [10,23]. We started the reference THz time-domain pulse *E*_ref_, the THz pulse from an empty setup purged with dry nitrogen, and, subsequently, the sample pulse *E*_sam_, the THz pulse transmitted through the sample from our experimental measurements. To construct the MAP estimator for the pulsed THz-TDS problem, the reference pulse (*E*_ref_) undergoes a filter *K_θ_* to parametrically simulate the effect of wave propagation through the sample, leading to a model-derived sample pulse (*E*_sam,model_). The optimization process involves tuning the filter parameters to minimize the difference between the experimental sample pulse (*E*_sam_) and the model-derived pulse (*E*_sam,model_) serving as the objective function. In our case, the chosen form of the filter, corresponding to the complex transmission coefficient obtained from Fresnel’s equation, is given by the following [10,24]:

(2) $$K_{\theta }\left(\omega \right)=\beta \frac{4n}{{\left(n+1\right)}^{2}}e^{i\frac{wd}{c}\;\left(n-1\right)\;-\;\frac{ad}{2}}\;,$$

where *d* is the thickness of the sample measured independently and *c* is the speed of light. *β* serves as an amplitude scaling factor compensating for multiple reflections of the signal, and additive noise is produced by detecting electronics and fluctuations in the optical pump laser power. This approach offers an easy noise modeling strategy that is not possible with the forward problem-solving in the frequency domain. As a result, we define this as a two-parameter modeling problem, with the optical parameters of interest, notably *n* and *α*, impacting the impulse transform. The THz imaging markers are then based on these two optimized parameters.

### 2.6. Tissue Block Preparation

For our studies, we used the LSL-KrasG12D/+Trp53L/LPtf1a-Cre (KPC) genetically modified mouse model to develop clinically relevant pancreatic ductal adenocarcinoma (PDAC) tumors for further investigation [10]. The KPC model is considered the gold standard for preclinical PDAC research because it developed tumors that closely resembled the diverse microenvironment found in human disease. Mice were euthanized after developing mature pancreatic tumors, and tumors were removed for formalin fixation and paraffin embedding (FFPE). The FFPE processing reduced the possibility of any substantial water content typically associated with highly vascular fresh tissue specimens. The tissue blocks (~4 mm thick) are then cut using a razor blade from large tissue cassettes, and THz-TDS imaging of those free-standing tissue blocks was performed in transmission geometry (as discussed in Section 2.4 and presented in Figure 4).

## 3. Results and Discussion

### 3.1. Bandwidth of the Spectra Obtained from Measured THz Transients

**Figure 5:**
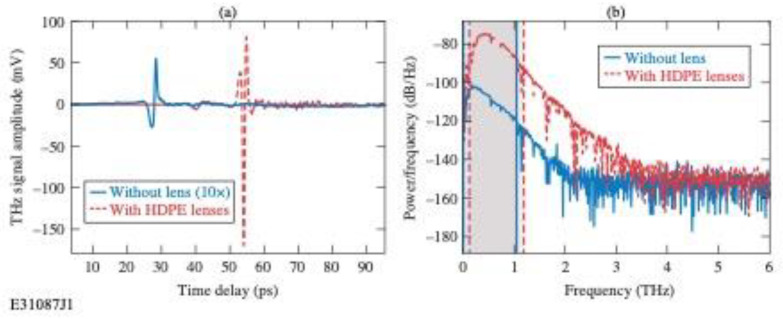
(**a**) THz transients using Teflon^TM^ lenses (blue solid line is multiplied 10× for better visibility) and HDPE lenses (red dashed line), measured under identical conditions without purging the system with dry nitrogen [hence, water signatures are visible in the FFTs shown in (**b**)]. (**b**) Fast Fourier transforms of time-domain waveforms shown in (**a**) with 10 dB bandwidths indicated.

To show the advantage of the insertion of HDPE lenses in our THz imaging and sensing setup, we performed comparative studies by measuring THz transients under the same conditions—an empty experimental setup in air environment—using the hemispherical Teflon^TM^ lenses and replacing them with the aspheric HDPE lenses. Figure 5a presents the time-domain waveforms of those signals, while Figure 5b shows their fast Fourier transforms (FFTs). As expected, because of their fine focusing and greater THz power transmission at the detector (see Figure 4b), the signal amplitude in Figure 5a with the HDPE lenses is significantly (about 30×) larger, most importantly, introducing HDPE lenses increased the central frequency from 0.02 to 0.43 THz (see Figure 5b) and the total bandwidth increased from ~2 THz to ~3.5 THz. The 10 dB bandwidth was also slightly enhanced and equal to 1.07 THz (see Figure 5b), causing the fractional bandwidth at the 10 dB figure of merit, defined as the 10 dB bandwidth divided by the central frequency value, to be as small as 2.5. The fractional bandwidth at 10 dB is the measure of the effective bandwidth in the focused region [25].

### 3.2. High-Resolution THz Imaging of Ex Vivo Murine Tissue Samples

**Figure 6:**
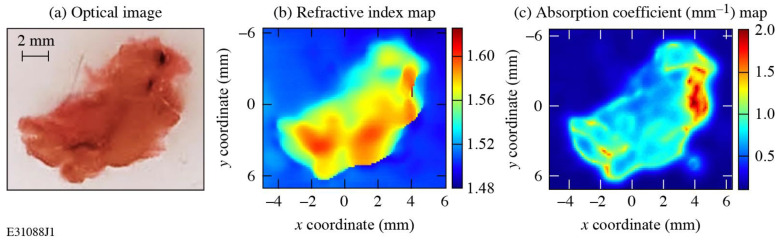
(**a**) An optical image of a 4 mm FFPE healthy murine tissue used in this study. High-resolution, 2D THz-imaging maps of the same tissue, using (**b**) the refractive index and (**c**) the absorption coefficient as imaging markers.

Figure 6a depicts an optical image of an FFPE healthy murine pancreas tissue, while Figure 6b,c show the corresponding high-resolution, 2D *n* and *α* parameter maps, respectively. The blue background regions in Figure 6b,c denote pure paraffin, and their *n* and *α* values are consistent with the THz-range pure paraffin data reported in the literature [26]. Note that tissue edges are very well resolved in both maps. The contrast of the *n* and *α* parameters within the tissue region reflects the inhomogeneous microenvironment of murine pancreas tissue [10]. Although water is believed to be the major cause of contrast in THz biomedical images, in this case, the FFPE tissues are fully dehydrated, so the contrast in the images occurs due to intrinsic biological variations in the tissue sample. The exact cause of the contrast can be analyzed through concurring histopathological studies, which was discussed in [10] and is beyond the scope of this work.

### 3.3. Quantitative Evaluation of R_2080_ Lateral Resolution

**Figure 7:**
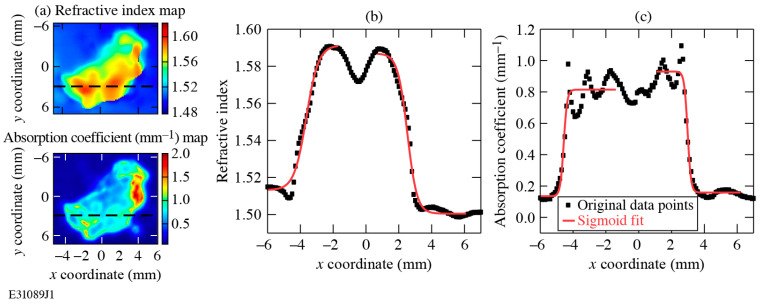
(**a**) The double-sigmoid fit function was applied to the line scans at *y* = 3 mm for both the *n* and *α* maps shown earlier in Figure 6. (**b**) Double-sigmoid fit for *n*. (**c**) Double-sigmoid fit for *α*.

Lateral resolution, also referred to as image resolution, is the most-significant element of maps and line scans. It represents the image produced when two structures lying alongside each other are perpendicular to the beam and is directly associated with the width of the THz beam. The resolution improves as the beam becomes narrower. In THz imaging, there is no commonly established method for determining the lateral resolution. Hence, we propose an estimation of the lateral resolution by evaluating the *R*_2080_ parameter. To quantify the lateral resolution of obtained THz images, especially at the paraffin/tissue boundaries, we focused on the *n* and *α* of single line scans, indicated in Figure 7a by black dashed lines. Figure 7a presents the same images as shown in Figure 6b,c. The line scans in Figure 7a are analyzed with the help of “sigmoid functions.” The features of the sigmoid functions are required to create models of state transitions in order to fit the data. The sigmoid function is utilized when only a transition from one state to another is required; the double-sigmoid function is used when the second state is transitory. Similarly, one can use a multi-sigmoid function for multiple-state transitions. The general form of a multi-sigmoid function is as follows:

(3) $$\theta \left(l\right)=\sum_{i=1}^{k}s_{i}\frac{1}{1+e^{-\left(1-c_{i}\right)/w_{i}}}\;,$$

where *l* is the scan position, *c_i_* denotes the location of the boundary, *w_i_* denotes the width of the transition from one region to another, *s_i_* denotes the relative strength of the corresponding transition within a single line scan, and *k* is the number of transitions present in each line scan. A physical transient-state scenario seldom exhibits symmetry, meaning each sigmoid should have a distinct transition period or width (*w_i_*) as it enters and exits the state. Since the above form of the sigmoid function introduces high computational complexity due to its exponential’s infinite asymptotic property for large positive arguments, we instead implemented a computationally simpler form that involves a hyperbolic tangent function [27]:

(4) $$\theta \left(1\right)=\sum_{i=1}^{k}s_{i}\frac{1}{2}\left[1+\tanh\left(\frac{1-c_{i}}{w_{i}}\right)\right]\;.$$

Since each dashed scan line in Figure 7a involves two transitions, one from the paraffin to the tissue and the second back from the tissue to the paraffin, we used the double-sigmoid fit with *k* = 2 in Equation (4). We developed a custom numerical code to perform the nonlinear least square fitting and calculate the values of *s_i_*, *w_i_*, and *c_i_* parameters. The fitted curves are shown in Figure 7b,c as solid red lines, while the back points are the *n* and *α* line scan data taken directly from our experimental images.

Next, we introduced the lateral resolution *R*_2080_ parameter, defined as the distance required for a 20–80% transition of a given biomarker at the boundary [28], as follows:

(5) $$R_{2080_{i}}=2\ln\left(4\right)\bar{w_{i}},$$

where $\bar{w_{i}}$ is an average value of *w*_1_ and *w*_2_ calculated from the sigmoid fit for each paraffin-to-tissue transition in the samples presented in Figure 7, respectively.

The *R*_2080_ parameters for the *n* and *α* maps calculated based on Equation (5) and the double-sigmoid fits shown in Figure 7 are presented in Table 1. It should be noted that our HDPE lenses, as discussed earlier in connection with Figure 4b, provide a diffraction-limited focus. Since the *R*_2080_ lateral resolution parameters are calculated from both the biomarkers across the 0.1–2.5-THz bandwidth, their spatial resolutions are also diffraction-limited, as well as limited by experimental variability in the markers between the paraffin and tissue regions. For comparison purposes, we also list in Table 1 the *R*_2080_ parameters for *n* and *α* maps collected using our earlier version of the THz-TDS setup [10], i.e., the one with a pair of hemispherical short-focal-length Teflon^TM^ lenses, placed next to the sample on both sides (see Figure 4a). Since the lower *R*_2080_ value indicates a sharper paraffin-tissue transition region, we note that implementation of the HDPE lenses significantly enhanced the resolution of our THz-TDS experimental system (by a factor of ~5 for *n* and ~2.6 for *α*).

## 4. Discussion

We presented a design scheme and, subsequently, the manufacturing and characterization process of cost-effective plano-aspheric focusing lenses for the high-resolution THz-TDS system in transmission geometry. We selected HDPE as a suitable lens material due to its inert nature, low cost, and low insertion loss within the usable THz bandwidth. We designed the lens considering our pre-existing optics in the THz transmission setup and manufactured the lens in the machine shop using a simple, computer-controlled lathe. The performance of the custom-made HDPE lenses was tested in our standard THz-TDS system by imaging a FFPE murine tissue block, which produced extremely well-resolved, high-resolution THz images of *n* and *α* biomarkers. We compared the performance of the aspheric HDPE lenses with our preexisting setup containing hemispherical Teflon^TM^ lenses. We also devised a lateral resolution calculation parameter, *R*_2080_, to estimate the lens impact on the THz image resolution and measured its lateral spatial resolution across the paraffin-tissue region to be ~300 µm for *n* and *α* THz images. This micrometer-scale lateral spatial resolution indicates that the error in the manufactured lens versus the ideal design did not affect the sensing resolution of our THz-TDS setup. Finally, we demonstrated via our design, and experimentally validated, that we could achieve diffraction-limited subwavelength spatial imaging resolution using these lenses. Additional detailed analyses of imaging markers at specific frequencies need to be carried out to further increase the sensing resolution of our THz-TDS imaging system.

## 5. Patents

We filed a US patent disclosure with the University of Rochester to obtain the intellectual property rights to this technology and the associated modeling of imaging biomarkers in the TH_Z_-TDS technique (US Patent Application #63/579,024).

## Figures and Tables

**Table 1:** *R*_2080_ spatial resolution parameters values for paraffin-tissue transition regions calculated from the sigmoid fits using *n* and α as imaging markers obtained from the time-domain scans.

Imaging Markers	*R*_2080_ (μm)	
	With HDPE Lenses	With TeflonTM Lenses
	Figure 4b	Figure 4a
	(100 μm Step Size)	(100 μm Step Size)
Refractive index *n*	333 (±19)	1850 (±38)
Absorption coefficient in mm^–1^ *α*	291 (±15)	765 (±17)

## Data Availability

The data presented in this study are available upon reasonable request from the corresponding author.
